# Folic acid ameliorates celecoxib cardiotoxicity in a doxorubicin heart failure rat model

**DOI:** 10.1080/13880209.2017.1299768

**Published:** 2017-03-08

**Authors:** Shafique Ahmad, Bibhu Prasad Panda, Kanchan Kohli, Mohammad Fahim, Kiran Dubey

**Affiliations:** a Department of Pharmacology, Faculty of Pharmacy, Jamia Hamdard, New Delhi, India;; b Department of Pharmacognosy and Phytochemistry, Faculty of Pharmacy, Jamia Hamdard, New Delhi, India;; c Department of Pharmaceutics, Faculty of Pharmacy, Jamia Hamdard, New Delhi, India;; d Department of Physiology, Hamdard Institute of Medical Sciences and Research, Jamia Hamdard, New Delhi, India

**Keywords:** Selective COX-2 inhibitor, cardiovascular, Tn-T, TNF-α

## Abstract

**Context:** The cardiotoxic effect of selective cyclo-oxygenase-2 inhibitors is well known. While rofecoxib and valdecoxib have been withdrawn, celecoxib remains on the market. Folic acid, a naturally occurring vitamin, has been shown to reduce myocardial ischemia and post-reperfusion injury in rats.

**Objective:** This study examined the cardiac effects of celecoxib and folic acid on doxorubicin-induced cardiomyopathy in rats.

**Materials and methods:** Cardiomyopathy was induced in male Wistar rats with six intraperitoneal injections of 2.5 mg/kg doxorubicin over a period of two weeks. The effect of 28 days of celecoxib (100 mg/kg/day) and its combination with folic acid (10 mg/kg/day) was studied on doxorubicin-induced cardiomyopathy according to serum lactate dehydrogenase (LDH), creatine kinase (CK-MB), troponin-T (Tn-T), tumor necrosis factor alpha (TNF-α), cardiac thiobarbituric acid reactive substance (TBARS), and glutathione (GSH) levels as well as systolic blood pressure (SBP), heart rate (HR) and ultrastructural studies.

**Results:** Celecoxib cardiotoxicity was manifested by significant increases in the LDH, Tn-T, TNF-α, CK-MB, SBP, HR (*p* < 0.001) and TBARS (*p* < 0.01) levels and a significant decrease in the GSH (*p* < 0.05) level when used alone or administered with doxorubicin. However, the combination of folic acid with celecoxib caused a significant reversal of these parameters and reduced the cardiotoxicity of celecoxib that was aggravated by doxorubicin. The ultrastructural study also revealed myocardial protection with this combination.

**Discussion and conclusion:** Folic acid protects against the cardiotoxic effects of celecoxib, which are aggravated in the presence of doxorubicin. Folic acid may act as a useful adjunct in patients who are taking celecoxib.

## Introduction

The potential adverse cardiovascular events reported with selective cyclooxygenase-2 (COX-2) inhibitors, rofecoxib, in the Vioxx Gastrointestinal Outcomes Research (VIGOR) study (Bombardier et al. 2000) and Adenomatous Polyp Prevention on Vioxx (APPROVe) trials (Bresalier et al. 2005) as well as with valdecoxib in the Coronary Artery Bypass Graft Surgery (CABG) trial (Nussmeier et al. 2005) led to their withdrawal from the market in 2004 and 2005, respectively. Etoricoxib was never approved by the United States Food and Drug Administration (FDA) because of safety concerns and the elevated risk of myocardial infarction (Andersohn et al. 2006). An increased risk of myocardial infarction has also been associated with the use of nonselective nonsteroidal anti-inflammatory drugs (NSAIDs), such as diclofenac, ibuprofen, and naproxen (Hippisley-Cox & Coupland 2005). Subsequently, the FDA labelled all COX-2 selective and nonselective NSAIDs as increasing the cardiovascular risk and asked the manufacturers of all marketed NSAIDs to revise their labelling with boxed warnings, highlighting the potentially serious cardiovascular events in patients with established ischemic heart disease (FDA 2005).

However, the FDA has allowed celecoxib (CEL) to remain on the market after concluding that the benefit of CEL outweighs the potential risk. The cardiovascular risk profile of CEL is much lower; most studies suggest the risk to be minor (McGettigan & Henry 2006). A differential effect of CEL has been reported on potassium and calcium ionic currents, which may account for its relative cardiovascular safety compared to rofecoxib or diclofenac (Brueggemann et al. 2009). Nevertheless, CEL may also cause a significant increase in serious cardiovascular events, such as myocardial infarction, stroke or congestive heart failure, as reported in the Adenoma Prevention with Celecoxib (APC) trial, which led to early discontinuation of the study (Solomon et al. 2005). Adjudicated serious cardiovascular events were also reported in 2.5% of subjects receiving CEL compared to 1.9% in the placebo group in the prevention of colorectal sporadic adenomatous polyps (Pre-SAP) trial (Arber et al. 2006).

The B-vitamin, folic acid (FA), long known for its role in preventing anaemia in expectant mothers and neural tube defects in newborns, has been shown to reduce myocardial ischemia and post-reperfusion injury in rats (Moens et al. 2008). Dietary FA supplementation has also been shown to diminish cardiac myocyte apoptosis in rats with streptozotocin-induced diabetes (Wu et al. 2008).

The interest in FA for treating cardiovascular disease stems from its critical role in converting homocysteine to methionine (Moat et al. 2004). FA deficiency has been associated with hyperhomocysteinemia, which has, in turn, been linked to a host of other cardiovascular diseases (Pandey et al. 2000). FA has also been found to improve endothelial function independent of its homocysteine-lowering effect in several clinical studies (Moens et al. 2007; Shirodaria et al. 2007).

Therefore, the present study investigates the cardiovascular effects of CEL along with FA in doxorubicin (DOX)-induced cardiomyopathy in rats. DOX cardiomyopathy, well documented in various animal models, is clinically relevant and mimics human syndrome of congestive heart failure (Siveski-Iliskovic et al. 1994; Chatterjee et al. 2010; Pontes et al. 2010).

## Materials and methods

### Drugs and chemicals

DOX was purchased as ADRIM injection manufactured by Fresenius Kabi Oncology Ltd (Pune, India). CEL and FA were procured as gift samples from Aurobindo Pharma Ltd (Hyderabad, India) and Tirupati Medicare Ltd (Paonta Sahib, India), respectively. The lactate dehydrogenase (LDH) (ENZOPAK) and creatine kinase (CK-MB) kits were purchased from Reckon Diagnostics Pvt. Ltd (Vadodara, India). ELISA Kits for estimations of Troponin-T (Tn-T) and tumor necrosis factor-alpha (TNF-α) were obtained from Cusabiotech Ltd (Wuhan, China), and Ray Biotech, Inc. (Norcross, USA), respectively. All other chemicals used in the experiment were of analytical grade. 

### Animals

Male albino Wistar rats, weighing 150 to 180 g, were procured from the Central Animal House Facility, Hamdard University, New Delhi, India. The animals were housed in polypropylene cages under standard laboratory conditions (12 h light/dark cycles) and had free access to a commercial pellet diet and water *ad libitum*. The animal house temperature was maintained at 25 ± 2 °C. The experimental protocol was approved by the Institutional Animal Ethics Committee and all studies were conducted under the guidelines of Committee for the Purpose of Control and Supervision of Experiments on Animals (CPCSEA).

### Experimental protocol

The animals were randomly divided into seven groups, each containing 10 animals, and were treated as follows:

**Table:** 

Group-I	Control: 0.5 mL/kg/d, 1% carboxymethylcellulose (CMC), p.o. for 28 days +0.5 mL/kg normal saline, intraperitoneal (i.p.), over a period of 2 weeks (3^rd^ and 4^th^ week)
Group-II	DOX: 2.5 mg/kg in six equal i.p. injections over a period of 2 weeks (total cumulative dose of 15 mg/kg)
Group-III	CEL: 100 mg/kg/d, p.o. suspended in 1% CMC for 28 days
Group-IV	CEL + DOX: 100 mg/kg/d, p.o for 28 days +2.5 mg/kg, six equal i.p. injections over a period of 2 weeks (3^rd^ and 4^th^ weeks)
Group-V	FA: 10 mg/kg/d, p.o. suspended in 1% CMC for 28 days
Group-VI	CEL + FA: 100 mg/kg/d, p.o. for 28 days +10 mg/kg/d, p.o. for 28 days
Group-VII	CEL + FA + DOX: 100 mg/kg/d, p.o. for 28 days +10 mg/kg/d, p.o. for 28 days +2.5 mg/kg in six equal i.p injections over a period of 2 weeks (3^rd^ and 4^th^ weeks)

The doses of CEL and FA used in the present study were selected based upon previous studies (Hagar 2002; Moens et al. 2008; Ashkavand et al. 2012; Buchineni et al. 2014). The hemodynamic parameters, such as the systolic blood pressure (SBP) and heart rate, were measured by tail-cuff plethysmography at baseline and then again after every week of drug administrations. After 24 h of the last dose administration, blood was withdrawn from the retro-orbital plexus under light ether anaesthesia for biochemical estimations of the serum LDH, CK-MB, Tn-T, and TNF-α. The rats were sacrificed and the hearts were rapidly removed, rinsed in ice-cold 0.9% sodium chloride solution, blotted and estimated for thiobarbituric acid reactive substance (TBARS) and glutathione (GSH). The cardiac tissue was also analyzed by transmission electron microscopy.

### Biochemical estimations in serum

#### LDH and CK-MB

The serum LDH and CK-MB activity were determined by kinetic methods using respective kits and UV spectrophotometer (Shimadzu). The LDH measurement was based on the principle that LDH catalyzes the oxidation of lactate to pyruvate, which is accompanied by a simultaneous reduction of NAD^+^ to NADH. The LDH activity in serum was proportional to the increase in absorbance due to the reduction in NAD^+^ at 340 nm (Bergmeyer 1974). The measurement of CK-MB was based on the principle that creatine kinase catalyzes the conversion of creatine phosphate to creatine; equimolar quantities of NADPH and creatine are formed at the same rate after the breakdown of glucose in this entire reaction. The photometrically measured rate of formation of NADPH was proportional to the CK activity at 340 nm (Rosalki 1967).

#### Tn-T and TNF-α

Tn-T and TNF-α were measured using rat-specific Tn-T (Cusabio) and TNF-α (RayBio) Elisa kits, respectively.

The microtiter plate provided in the Tn-T kit had been pre-coated with an antibody specific to Tn-T. Standards or samples were then added to the appropriate microtiter plate wells with a biotin-conjugated antibody preparation that was specific for Tn-T, and avidin conjugated to horseradish peroxidase (HRP) was added to each microplate well and incubated. Then, a 3,3′,5,5′-tetramethylbenzidine (TMB) substrate solution was added to each well. Only those wells that contained Tn-T, biotin-conjugated antibody and enzyme-conjugated Avidin exhibited a change in color. The enzyme-substrate reaction was terminated by the addition of a sulfuric acid solution, and the color change was spectrophotometrically measured at a wavelength of 450 ± 2 nm. The concentration of Tn-T in the samples was then determined by comparing the optical density of the samples to the standard curve.

The TNF-α assay employed an antibody specific for rat TNF-α coated on a 96-well plate. Standards and samples were pipetted into the wells and TNF-α present in the samples were bound to the wells by the immobilized antibody. The wells were washed, and biotinylated anti-rat TNF-α antibody was added. After washing away unbound biotinylated antibody, HRP-conjugated streptavidin was pipetted into the wells. The wells were again washed, TMB substrate solution was added to the wells and color was developed in proportion to the amount of bound TNF-α. The Stop Solution changed color from blue to yellow, and the intensity of the color was measured at 450 nm.

### Biochemical estimations in cardiac tissue

#### TBARS

Lipid peroxidation was estimated by measuring the levels of TBARS that were measured as nanomoles of malondialdehyde in cardiac tissues by the method of Ohkawa et al. (1979). The pink chromogen produced by the reaction of the thiobarbituric acid with malondialdehyde, a secondary product of lipid peroxidation, was estimated at 540 nm.

#### GSH

GSH was estimated by the Ellman method, i.e., 5,5′-dithiobis-2-nitrobenzoic acid (DTNB), is reduced by –SH groups to form one mole of 2-nitro-5-mercaptobenzoic acid per mole of –SH. The nitro mercaptobenzoic acid anion has an intense yellow color that was spectrophotometrically determined at 412 nm (Sedlak & Lindsay 1968) and proteins were determined using bovine serum albumin as a standard. Protein reacts with Folin Ciocalteau reagent to yield a colored complex, the absorbance of which was spectrophotometrically determined at 750 nm (Lowry et al. 1951).

### Hemodynamic parameters

#### SBP and heart rate

The rats were trained for two weeks in Perspex restrainers for measurement of the blood pressure and heart rate using noninvasive techniques before experimentation. The SBP and heart rate recordings as beats per min (BPM) were measured on conscious rats by tail-cuff plethysmography using LE 5000, Storage Pressure Meter, Letica (USA) at baseline (0 week) and then again after the 1st, 2nd, 3rd and 4th weeks of drug administration (Molina et al. 1994).

### Transmission electron microscopy

Samples were fixed in modified Karnovasky’s fluid, buffered with 0.1 M sodium phosphate for 10–18 h and then postfixed for 2 h in 1% osmium tetroxide in the same buffer at 4 °C. After several washes in 0.1 N sodium phosphate buffers, the specimens were dehydrated in graded acetone solutions and embedded in CY212. Ultrathin sections of 60-80 nm were stained in alcoholic urbanely acetate and lead citrate before examining the grids with a transmission electron microscope TECNAI 200 kV TEM (Fei, Electron Optics, Hillsboro, USA). 

### Statistical analysis

Statistical analysis was performed using Graph pad 3.0 (Graph pad software; San Diego, CA). All data are expressed as a mean ± standard deviation. One-way analysis of variance (ANOVA) was used to compare the various parameters among groups followed by *post hoc* comparison using the Tukey–Kramer multiple comparisons test. Repeated-measures ANOVA (Two-way ANOVA) was used to observe the change in each group, which was followed by *post hoc* comparison using Tukey–Kramer test, and *p* < 0.05 was considered as significant.

## Results

### Biochemical estimations in serum

#### Effect on LDH and CK-MB

Table 1 shows the effect of CEL and its combination with FA on serum LDH and CK-MB.

**Table 1.** Effect of CEL and its combination with FA on the LDH and CK-MB levels.

Group	Treatment	LDH (IU/L)	CK-MB (IU/L)
I	CONTROL	83.67 ± 4.32	67.17 ± 8.07
II	DOX	213.34 ± 12.06***	181.20 ± 8.02***
III	CEL	105.25 ± 13.34†††	91.02 ± 9.91†††
IV	CEL + DOX	249.60 ± 11.65###	202.67 ± 17.70###
V	FA	87.63 ± 4.68	75.98 ± 4.42
VI	CEL + FA	91.18 ± 2.54*	73.79 ± 6.18*
VII	CEL + FA + DOX	235.33 ± 9.83†	186.60 ± 5.99†

Values are expressed as the mean ± SD and analyzed by one-way ANOVA followed by the Tukey–Kramer multiple comparison test.

* *p* < 0.05 (VI vs. III).

† *p* < 0.05 (VII vs. IV).

*** *p* < 0.001 (II vs. I).

††† *p* < 0.001(III vs. I)

### *p* < 0.001(IV vs. II), DOX: Doxorubicin; CEL: Celecoxib; FA: Folic acid; DOX: Doxorubicin.

The serum LDH and CK-MB levels were significantly increased (*p* < 0.001) in the DOX (Group II) treated group compared to the control group. Treatment with CEL (Group III) significantly increased the LDH and CK-MB levels (*p* < 0.001) compared to the control group. There was a significant increase in the LDH and CK-MB levels (*p* < 0.001) when CEL was administered with DOX (Group-IV) compared to the DOX treatment (Group-II). The combination of FA with CEL (Group-VI) significantly decreased the LDH and CK-MB levels (*p* < 0.05) compared to CEL alone (Group-III). A significant (*p* < 0.05) decrease in the LDH and CK-MB levels was also seen when FA was administered in combination with CEL + DOX (Group-VII) compared to the CEL + DOX treated group (Group IV). However, treatment with FA alone for 28 days did not show a significant change in the LDH and CK-MB levels compared to the control.

#### Effect on Tn-T and TNF-α

Table 2 shows the effect of CEL and its combination with FA on Tn-T and TNF-α.

**Table 2.** Effect of CEL and its combination with FA on the Tn-T and TNF-α levels.

Group	Treatment	Tn-T (pg/ml)	TNF-α (pg/ml)
I	CONTROL	10.89 ± 0.94	0.044 ± 0.01
II	DOX	196.09 ± 26.58***	1740.91 ± 148.68***
III	CEL	138.27 ± 11.92†††	1109.67 ± 91.71†††
IV	CEL + DOX	389.77 ± 70.11###	2743.78 ± 188.27###
V	FA	9.57 ± 0.81	0.042 ± 0.01
VI	CEL + FA	68.17 ± 12.08**	683.86 ± 115.47§§§
VII	CEL + FA + DOX	204.55 ± 18.71‡‡‡	1892.41 ± 244.61‡‡‡

Values are expressed as the mean ± SD and analyzed by one-way ANOVA followed by the Tukey–Kramer multiple comparison test.

** *p* < 0.01 (VI vs. III).

*** *p* < 0.001 (II vs. I).

††† *p* < 0.001(III vs. I).

### *p* < 0.001 (IV vs. II).

§§§ *p* < 0.001 (VI vs. III), and

‡‡‡ *p* < 0.001 (VII vs. IV).

CEL: Celecoxib; FA: Folic acid; DOX: Doxorubicin.

The Tn-T and TNF-α levels were significantly increased (*p* < 0.001) in the DOX (Group II) treated group compared to the control group. Treatment with CEL (Group III) significantly raised the Tn-T and TNF-α levels (*p* < 0.001) compared to the control. There was a significant increase in the Tn-T and TNF-α levels (*p* < 0.001) when CEL was administered with DOX (Group-IV) compared to the DOX treatment (Group-II). The combination of FA with CEL (Group-VI) significantly decreased the Tn-T (*p* < 0.01) and TNF-α (*p* < 0.001) levels compared to CEL treatment alone (Group-III). A significant decrease in Tn-T and TNF-α (*p* < 0.001) was also observed when FA was administered in combination with CEL + DOX (Group-VII) when compared to CEL + DOX treatment (Group IV). However, treatment with FA alone for 28 days did not show a significant change in the Tn-T and TNF-α levels compared to the control.

### Biochemical estimations in cardiac tissue

Table 3 shows the effect of CEL and its combination with FA on cardiac TBARS and GSH as markers of lipid peroxidation.

**Table 3.** Effect of CEL and its combination with FA on the cardiac TBARS and GSH levels.

Group	Treatment	TBARS (nmMDA/mg protein)	GSH (μg/mg of protein)
I	CONTROL	2.24 ± 0.55	22.36 ± 2.39
II	DOX	8.31 ± 2.61***	10.63 ± 1.59***
III	CEL	4.89 ± 0.83**	18.74 ± 2.43*
IV	CEL + DOX	12.32 ± 2.28†††	8.22 ± 1.76
V	FA	1.95 ± 0.57	23.25 ± 2.52
VI	CEL + FA	2.51 ± 0.63†	20.54 ± 2.44
VII	CEL + FA + DOX	9.54 ± 1.25††	17.09 ± 1.55###

Values are expressed as the mean ± SD and analyzed by one-way ANOVA followed by the Tukey–Kramer multiple comparison test.

* *p* < 0.05(III vs. I).

† *p* < 0.05 (VI vs. III).

** *p* < 0.01(III vs. I).

†† *p* < 0.01 (VII vs. IV).

*** *p* < 0.001 (II vs. I).

††† *p* < 0.001 (IV vs. II), and

### *p* < 0.001(VII vs. IV).

CEL: Celecoxib; FA: Folic acid; DOX: Doxorubicin.

#### Effect on TBARS and GSH

The TBARS levels were significantly increased (*p* < 0.001) and that of GSH was significantly decreased (*p* < 0.001) in the DOX (Group II) treated group compared to the control. Treatment with CEL (Group III) significantly increased the TBARS (*p* < 0.01) and decreased the GSH (*p* < 0.05) compared to the control (Group I). The combination of FA with CEL (Group-VI) significantly decreased the TBARS level (*p* < 0.05) compared to CEL treatment alone (Group-III). There was a significant increase (*p* < 0.001) in TBARS when CEL was administered with DOX (Group-IV) compared to the DOX-treated group (Group-II). There was no significant change in GSH when the CEL + DOX (Group-IV) treatment was compared to the DOX treatment (Group-II) or when the combination of FA with CEL (Group-VI) was compared to the CEL treatment alone (Group-III). A significant decrease in TBARS (*p* < 0.01) and an increase in GSH (*p* < 0.001) was observed when FA was administered in combination with CEL + DOX (Group-VII) compared to the CEL + DOX treated group (Group IV). However, treatment with FA alone for 28 days did not show a significant change in the TBARS or GSH level compared to the control.

### Hemodynamic parameters

#### SBP

**Figure 1.**
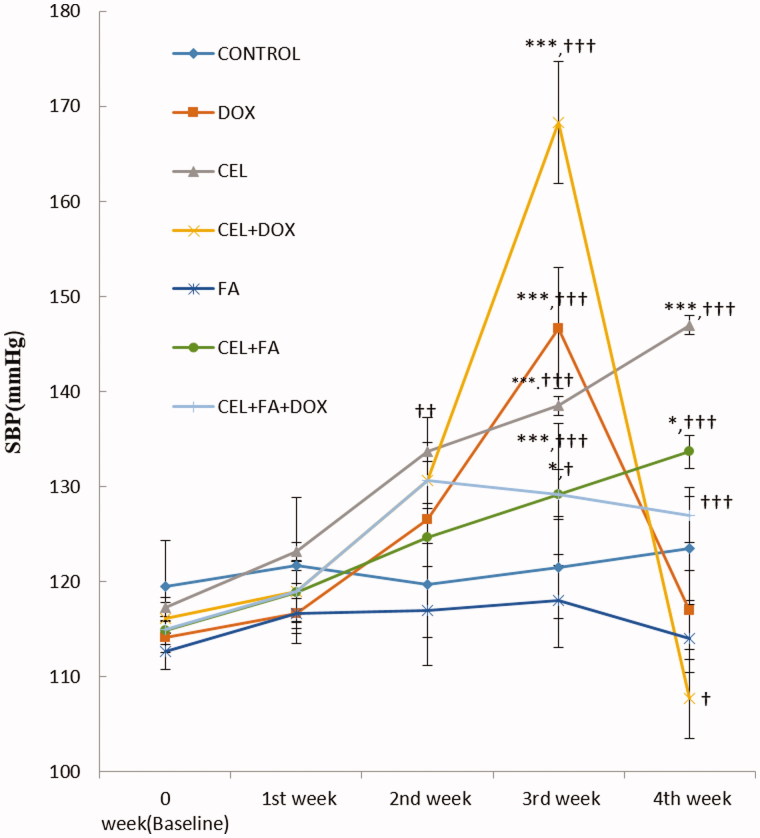
Effect of CEL and its combination with FA on SBP. *p < 0.05 CEL + FA (3rd and 4th weeks) vs. Baseline, ***p < 0.001 CEL (3rd and 4th weeks) vs. Baseline; DOX (3rd week) vs. Baseline; and CEL + DOX (3rd week) vs. Baseline. †*p* < 0.05 CEL + FA (3rd week) vs. CEL, CEL + DOX (4th week) vs. DOX; ††*p* < 0.01 CEL (2nd week) vs. Control, †††*p* < 0.001 CEL (3rd and 4th weeks) vs. Control; CEL + FA (4th week) vs. CEL; DOX (3rd week) vs. Control; CEL + DOX (3rd week) vs. DOX; and CEL + FA + DOX (3rd and 4th weeks) vs. CEL + DOX. DOX: Doxorubicin; CEL: Celecoxib; FA: Folic acid; SBP: systolic blood pressure.

The effect of CEL and its combination with FA on SBP in conscious rats is depicted in Figure 1.

The control group treated with 1% CMC and the group treated with FA alone (group-V) showed no significant change in SBP during four weeks of treatment. Treatment with CEL for four weeks significantly increased the SBP (*p* < 0.001) at the end of 3rd and 4th weeks compared to baseline and the control. Although a significant increase in the SBP (*p* < 0.05) was also observed when CEL was administered with FA (Group-VI) at the end of 3rd and 4th weeks of treatment compared to baseline, there was a significant decrease at the end of the 3rd week (*p* < 0.05) and a highly significant decrease in the SBP at the end of the 4th week (*p* < 0.001) when compared to CEL treatment alone. There was a significant increase in the SBP (*p* < 0.001) during the 1st week of treatment with DOX (which was administered from the 3rd week), which returned to baseline after two weeks of treatment. The SBP was also significantly increased at the end of the 3rd week (*p* < 0.001), but it was decreased at the end of the 4th week when CEL was administered with DOX (group IV) compared to the DOX-treated group (Group II). However, the combination of FA with CEL + DOX (Group-VII) caused a highly significant decrease (*p* < 0.001) and highly significant increase (*p* < 0.001) in the SBP at the end of the 3rd and 4th weeks when compared to the CEL + DOX treatment (Group IV).

### Heart rate

**Figure 2.**
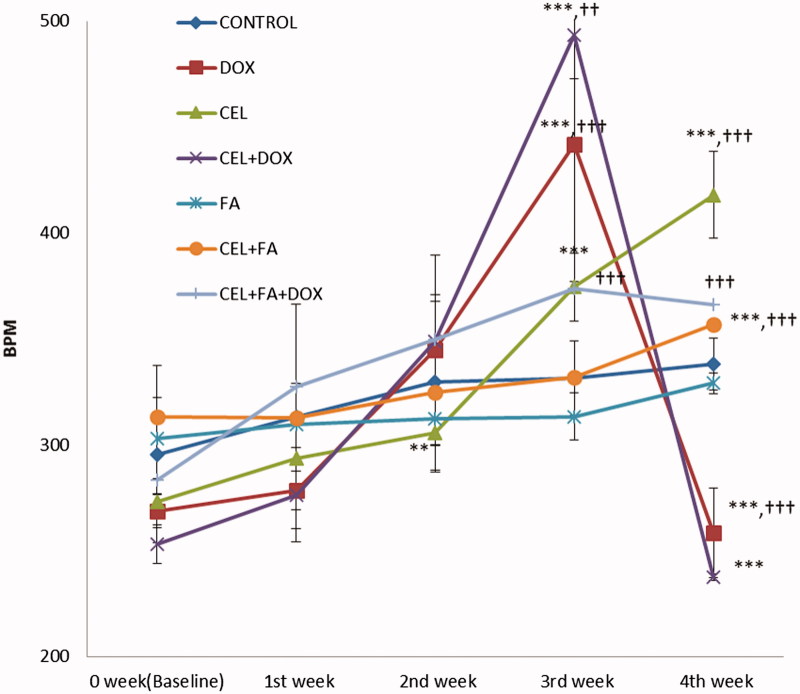
Effect of CEL and its combination with FA on heart rate. **p < 0.01 CEL (2nd week) vs. Baseline, ***p < 0.001 CEL (3rd and 4th weeks) vs. Baseline; CEL + FA (4th week) vs. Baseline; DOX (3rd week) vs. baseline; DOX (3rd vs. 4th week); CEL + DOX (3rd week) vs. Baseline; and CEL + DOX (3rd vs. 4th week). ††*p* < 0.01 CEL + DOX (3rd week) vs. DOX, †††*p* < 0.001 CEL (4th week) vs. Control; CEL + FA (4th week) vs. CEL; DOX (3rd and 4th weeks) vs. Control; and CEL + FA + DOX (3rd and 4th weeks) vs. CEL + DOX. DOX: Doxorubicin; CEL: Celecoxib; FA: Folic acid; BPM: Beats per min.

The effect of CEL and its combination with FA on the heart rate as BPM in conscious rats is depicted in Figure 2.

The control group treated with 1% CMC or FA showed no significant change in BPM during four weeks of treatment. Treatment with CEL alone (Group III) significantly increased the beats per min during the 2nd week (*p* < 0.01) and then again after the 3rd and 4th weeks of treatment (*p* < 0.001) compared with baseline. The increase in BPM with CEL was also highly significant (*p* < 0.001) when compared to the 2nd, 3rd and 4th weeks of treatment. The combination of FA with CEL caused no significant change during the 1st, 2nd and 3rd weeks of treatment, but it caused a significant decrease in the BPM (*p* < 0.001) at the end of the 4th week of treatment compared to CEL treatment alone. There was a significant increase in the BPM (*p* < 0.001) during the 1st week of treatment with DOX (which was administered from the 3rd week) compared with baseline, which was followed by a significant decrease (*p* < 0.001) at the end of the 4th week compared with the 3rd week. Similarly, when CEL was administered with DOX (Group-IV), the BPM significantly increased (*p* < 0.001) at the end of the 3rd week, which was followed by a significant (*p* < 0.001) decrease at the end of the 4th week compared with the 3rd week. The combination of CEL with DOX caused a significant increase in the BPM (*p* < 0.01) at the end of the 3rd week and a non-significant decrease at the end of the 4th week compared to DOX treatment. However, the combination of FA with CEL + DOX (Group VII) caused a highly significant decrease in the BPM (*p* < 0.001) at the end of the 3rd week and a highly significant increase (*p* < 0.001) at the end of the 4th week compared with the CEL + DOX treatment (Group IV).

### Transmission electron microscopy

**Figure 3.**
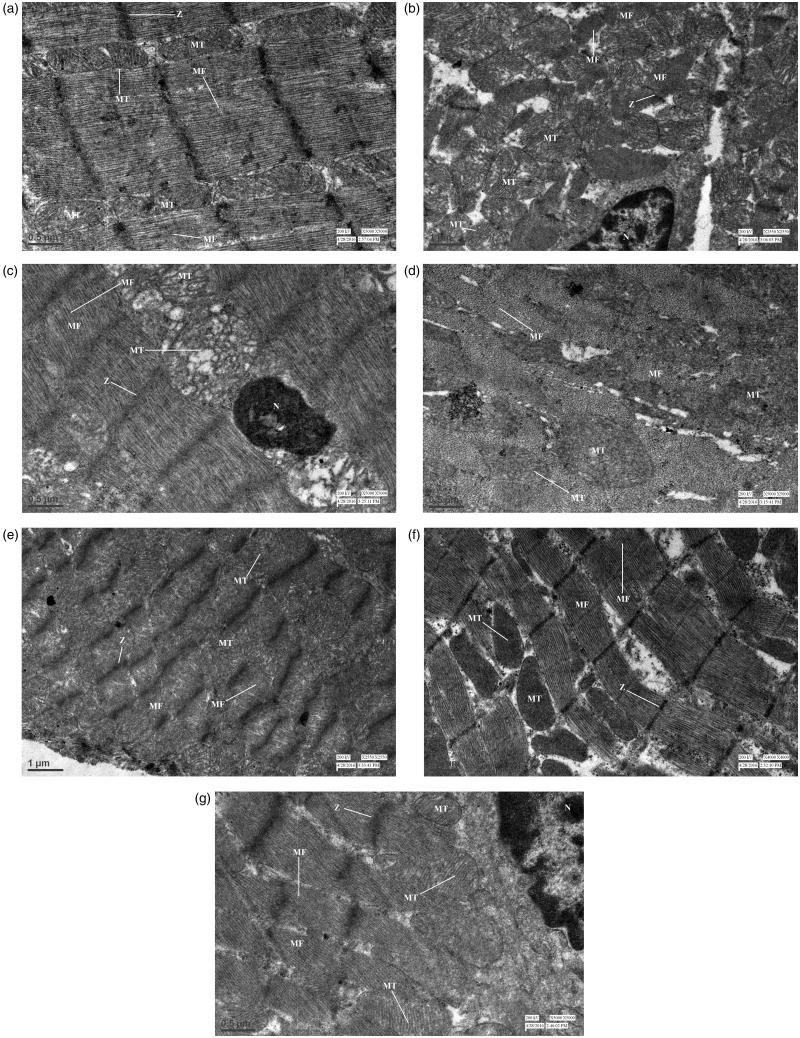
Transmission electron microscopy of cardiomyocytes. (a) Normal control group showing well-defined Z-bands (Z), myofibrils (MF) arranged in regular rows and mitochondria (MT). (b) DOX-treated group showing disarray of the myofibrillar arrangement, swollen mitochondria, and a pyknotic nucleus (N) with chromatin margination. (c) CEL-treated group showing loss of myofibrils, mild mitochondrial swelling, and condensed chromatin in the nucleus. (d) CEL + DOX treated group showing severe disruption revealing loss of myofibrils and mitochondrial swelling. (e) FA-treated group showing well-defined Z-Bands, myofibrils, and mitochondria. (f) CEL + FA-treated group showing normal myofibrils, well-defined Z-Bands, and mitochondria. (g) DOX + CEL + FA-treated group showing maintenance of myofibrillar arrangement with mild mitochondrial swelling and margination of chromatin in the nucleus (N). DOX: Doxorubicin; CEL: Celecoxib; FA: Folic acid.

Figure 3(a–g) shows electron micrographs of cardiomyocytes in various treatment groups. The electron micrographs of cardiomyocytes of rats treated with 1% CMC (Figure 3(a)) and FA alone (Figure 3(e)) showed a normal ultrastructural appearance of myofibrils with well-defined Z bands arranged in regular rows and mitochondria between the myofibrils. The cardiomyocytes of the group treated with CEL alone (Figure 3(c)) showed disruption of the ultrastructural appearance with loss of myofibrils, mild mitochondrial swelling, and a nucleus with condensed chromatin. However, the cardiomyocytes of the group treated with FA and CEL (Figure 3(f)) showed a normal ultrastructural appearance of myofibrils with well-defined Z bands and mitochondria in between the myofibrils. The cardiomyocytes of the group treated with DOX (Figure 3(b)) showed disarray of the myofibrillar arrangement, swollen mitochondria in clusters, and margination and clumping of chromatin in the pyknotic nucleus. The group treated with CEL and DOX (Figure 3(d)) showed severe disruption of the ultrastructural appearance, revealing loss of myofibrils and mitochondrial swelling. However, the group administered FA in combination with CEL and DOX (Figure 3(g)) revealed maintenance of the myofibrillar arrangement with mild mitochondrial swelling and margination of chromatin in the nucleus.

## Discussion

The present study explores the cardiovascular effects of CEL along with FA in the normal myocardium as well as in the presence of cardiac failure induced by DOX in Wistar rats. DOX has been used at a dose level of 2.5 mg/kg in six equal intraperitoneal injections, as reported previously (Siveski-Iliskovic et al. 1994; Luo et al. 1999; Li et al. 2000). The repeated administrations of DOX at this dose level produces delayed, progressive, and chronic cardiotoxicity with myocardial lesions that are similar to those reported in humans (Pontes et al. 2010). DOX-induced cardiomyopathy was manifested by significant increases in the serum LDH, CK-MB, Tn-T and TNF-α levels as markers of cardiac damage. The DOX-induced cardiomyopathy also caused a significant increase in TBARS and a significant decrease in GSH, which can act as markers of oxidative stress. Similar increases in serum levels of LDH, CK-MB, TNF-α, Tn-T, and cardiac TBARS, and decreases in cardiac GSH have been reported with DOX-induced cardiomyopathy (Herman et al. 1998; Mohamed et al. 2004; Jia et al. 2014).

We found that the administration of 100 mg/kg/d of CEL caused cardiotoxicity in rats, as manifested by highly significant increases in the serum LDH, CK-MB, Tn-T and TNF-α levels. The concomitant administration of FA with CEL caused significant decreases in the LDH, CK-MB, Tn-T, TNF-α and TBARS levels without affecting the GSH level. Similarly, CEL also aggravated DOX-induced cardiomyopathy, as shown by the significant increases in all of these biochemical markers and significant reversal of these parameters when administered in combination with FA. The ultrastructural studies showed loss of myofibrils, mitochondrial swelling, and condensed chromatin in the nuclei of cardiomyocytes in the CEL treated group, whereas the group treated with FA in combination with CEL revealed a normal ultrastructural appearance of myofibrils with well-defined Z bands that were arranged in regular rows with the nucleus and mitochondria in between the myofibrils. CEL aggravated cardiotoxicity of DOX was demonstrated by severe disruption of the ultrastructural appearance, indicating loss of myofibrils, mitochondrial swelling and pronounced margination of chromatin in the nucleus. However, the group given FA in combination with CEL and DOX revealed maintenance of a myofibrillar arrangement with only mild mitochondrial swelling and margination of chromatin in the nucleus.

These data show that the administration of CEL produced cardiotoxicity in both the presence and absence of preexisting heart failure and that the concomitant administration of FA ameliorated the cardiotoxicity produced by CEL in both of these conditions.

Four weeks of treatment with CEL significantly raised the SBP by approximately 16, 21 and 30 mm Hg and the heart rates were 33, 102, and 145 BPM during the 2nd, 3rd and 4th weeks of treatment, respectively, compared to baseline values. The concomitant administration of FA with CEL caused a significant decrease in the SBP during the 3rd and 4th weeks of treatment compared to the group that was treated with CEL, although treatment with FA alone for 4 weeks did not produce significant changes in the SBP. Selective COX-2 inhibition with CEL has been shown to elevate the blood pressure and increase leukocyte adherence to the endothelium in both normal and hypertensive rats (Muscará et al. 2000). FA has been found to prevent and reverse adrenocorticotropic hormone-induced hypertension in rats (Miao et al. 2007). While the Celecoxib Rofecoxib Efficacy and Safety in Comorbidities Evaluation Trial (CRESCENT) did not show an increase in the SBP after 6 weeks of therapy (Sowers et al. 2005), the CLASS trial found a relatively low, non-dose dependent incidence of hypertension with CEL compared with diclofenac and ibuprofen (Silverstein et al. 2000). Whelton et al. (2000) found a 1.6–3% incidence of oedema with varying doses of CEL, which is similar to the incidence found with the use of other NSAIDs.

All coxibs induce salt and water retention, leading to oedema and worsening hypertension, as do conventional NSAIDs. They can also cause an acute decline in renal function and the glomerular filtration rate due to inhibition of constitutively expressed COX-2 in the kidneys (Rios et al. 2012).

NSAIDs impair renal function and cardiac homeostasis in patients with preexisting heart failure; they can therefore increase the risk of heart failure in such high-risk patients or patients with established cardiovascular disease (Bleumink et al. 2003). DOX, which causes free radical-mediated cardiac decompensation in humans, has been used in the present study to assess the effect of selective COX-2 inhibition with CEL in rats with cardiac injury to match typical clinical practice, especially in primary care.

It is now known that COX-2 is expressed during the late phase of ischemic preconditioning and protects against both myocardial stunning and myocardial infarction (Shinmura et al. 2000). This cardio protective effect of COX-2 is mediated by the synthesis of PGE_2_ and PGI_2_ (Bolli et al. 2002). NSAIDs, especially COX-2 inhibitors, may abrogate COX-2 dependent late preconditioning and augment cell death and dysfunction by preventing the adaptive response of the heart to stress. The results of our study also indicate that the DOX-induced cardiotoxicity was aggravated in the presence of a selective COX-2 inhibitor, CEL.

In our study, four weeks of treatment with CEL significantly increased the heart rate when compared to baseline and when compared to each week of treatment.

CEL has been shown to modulate ion channel currents, produce arrhythmic beating of *Drosophila* hearts (Frolov et al. 2008) and inhibit hERG potassium channels expressed in HEK-293 and CHO cells, which play a significant role in causing drug-induced QT prolongation and cardiac arrhythmias (Frolov et al. 2011). The combination of FA with CEL prevented increases in the heart rate during four weeks of treatment in the present study. FA and vitamin B_12_ supplementation have been shown to attenuate isoprenaline-induced myocardial infarction and tachycardia in experimental hyperhomocysteinemic rats (Hagar 2002).

The adverse thrombotic events observed with COX-2 inhibitors is due to selective inhibition of prostacyclin by these agents, leading to unopposed thromboxane action (Mukherjee & Topol 2003); thrombosis would be more likely to occur in patients who are already at increased risk because of other underlying conditions. In fact, the arterial thrombosis occurred after the initiation of CEL in 4 patients with lupus anticoagulant (Crofford et al. 2000).

The risk associated with selective COX-2 inhibitors could be obliterated by the presence of other endothelium-derived substances, such as nitric oxide, carbon monoxide, and CD-39 that protect against thrombosis (Marcus et al. 2002). The prostacyclin depression secondary to COX-2 blockade may be combatted by up-regulation of nitric oxide-induced vasodilation (Segev & Katz 2004). ‘The recognition that COX-2 is an obligatory co-mediator together with inducible nitric oxide synthase (iNOS), of the protection afforded by late preconditioning, has implications for the clinical use of COX-2 selective inhibitors as well as nonselective COX inhibitors’ (Bolli et al. 2002). Therefore, the cardiovascular risk may be minimized in patients who receive NSAIDs along with a nitric oxide donor. A cheaper alternative to a nitric oxide donor would be FA or its metabolite, 5-methyl tetrahydrofolate, which has been shown to improve nitric oxide production and prevent superoxide generation via uncoupling of nitric oxide synthase by stabilizing its cofactor, tetrahydrobiopterin (Antoniades et al. 2006). Several other studies have reported that FA, or its active metabolite, improves endothelial function (Förstermann & Münzel 2006; Moat et al. 2006). A high dose of 30 mg of FA has been found to increase adenosine-stimulated myocardial blood flow in patients with coronary artery disease while simultaneously acutely lowering arterial pressure (Tawakol et al. 2005). Oster demonstrated that long-term FA treatment at a dose of 40–80 mg/d, which is far higher than the typical dose, for 10 years reduced the incidence of myocardial infarction, angina pectoris, and the requirement for nitroglycerin in patients with coronary artery disease (McCarty 2007). A meta-analysis of randomized controlled trials of FA supplementation has not shown any risk reduction or all-cause mortality among patients with a history of cardiovascular or renal disease (Bazzano et al. 2006). However, the dose of FA in the intervention groups, among trials included in this study was low and ranged from 0.5 to 15 mg/day.

In the present study, we used a high dose of 10 mg/kg/d FA, which has been shown to prevent myocardial ischaemia and heart failure in Wistar rats *in vitro* and *in vivo* (Hagar 2002; Moens et al. 2008). This dose corresponds to the human equivalent dose of approximately 113 mg for a 70-kg person (Reagan-Shaw et al. 2007). The use of a high-dose FA of at least 200 mg per subject or an equivalent dose of a FA derivative has been claimed to blunt myocardial dysfunction during ischemia and to ameliorate post-reperfusion injury (Moens 2009). However, proper clinical trials are required to establish safety and efficacy of a mega dose of FA in humans that may be administered concurrently with other NSAIDs.

## Conclusions

This study showed that CEL was cardiotoxic and aggravated DOX cardiotoxicity in Wistar rats. FA prevented CEL cardiotoxicity in the normal and injured myocardium of rats. FA may be a useful adjunct in patients who are prescribed CEL.

## Geolocation information

The study was carried out at Jamia Hamdard, Hamdard University, New Delhi.

The geolocation of the study area is 28.5154° latitude and 77.2521° longitude.
